# Additions to the Knowledge of the Genus *Pezicula* (Dermateaceae, Helotiales, Ascomycota) in China

**DOI:** 10.3390/biology11101386

**Published:** 2022-09-23

**Authors:** Huan-Di Zheng, Wen-Ying Zhuang

**Affiliations:** State Key Laboratory of Mycology, Institute of Microbiology, Chinese Academy of Sciences, Beijing 100101, China

**Keywords:** fungi, Leotiomycetes, new taxa, phylogeny, species diversity

## Abstract

**Simple Summary:**

Species of the genus *Pezicula* are endophytes, phytopathogens, or saprobes, and some of them have biocontrol potential, promoting plant growth and resistance to environmental stress. The species diversity of *Pezicula* in China was reviewed. Two new species (*P. ellipsoids* and *P. fusispora*) and two new Chinese records (*P. acericola* and *P. carpinea*) were discovered using morphological and molecular approaches. Descriptions and illustrations of macroscopic and microscopic features were provided for the new and newly found taxa. *Pezicula aurantiaca* was excluded from the fungal catalogue of China. Together with the previously reported taxa (*P. cinnamomea*, *P. ericae*, *P. heterochroma*, *P. magnispora*, *P. melanigena*, *P. neosporulosa*, *P. ocellata*, *P. rhizophila*, *P.* cf. *rubi*, and *P. subcarnea*), 14 *Pezicula* species are currently known from China. The results provide updated information and improve our understanding of the genus.

**Abstract:**

We describe two new species of *Pezicula* (Dermateaceae, Ascomycota), *P. ellipsoides* and *P. fusispora*, which are discovered in China. *Pezicula ellipsoides* sp. nov. is distinct in producing 2–3 stipitate apothecia on a basal stroma with a light yellow hymenium, broadly ellipsoid 0–1(–2)-septate ascospores, and divergent DNA sequence data. *Pezicula fusispora* sp. nov. is characterized by sessile apothecia, 0.3–0.8 mm in diam, a yellowish hymenium, J+ asci 135–170 × 15–21 μm, multiseptate ascospores, 33–48 × 7–10.3 μm, and growing on rotten bamboo. In addition, *Pezicula acericola* and *P. carpinea* are reported for the first time from China, and 11 *Pezicula* species previously reported from China are reviewed and briefly noted, of which one was a misidentified species. Phylogenetic analyses inferred from ITS nrDNA sequences confirm the placement of *P. ellipsoides* and *P. cinnamomea* in the genus *Pezicula*.

## 1. Introduction

*Pezicula* Tul. & C. Tul. belongs to the family Dermateaceae Fr. (Helotiales, Leotiomycetes), which was established in 1869 and typified by *P. carpinea* (Pers.) Tul. ex Fuckel [1]. The sexual stages of the genus are often found as bright-colored, fleshy, pruinose or pulverulent, sessile to short-stipitate apothecia on woody substrates [2]. The asexual stages of *Pezicula* were originally placed in *Cryptosporiopsis* Bubák & Kabát, which is treated as a later synonym according to the ‘one fungus–one name’ rule of the International Code of Nomenclature for algae, fungi, and plants [3]. *Pezicula* species are endophytes, phytopathogens, or saprobes, and some of them have the abilities of antagonizing plant pathogens, producing compounds with antimicrobial activities, promoting plant growth, and increasing plant resistance to environmental stress [2,4,5,6,7]. Several regional investigations of the genus have been carried out [8,9,10,11]. A monographic treatment of *Pezicula* was published by Verkley in 1999, in which 26 species were accepted [2]. Eleven taxa were further introduced, ten species were transferred to *Pezicula* from other genera, and one name was treated as synonym thereafter [3,5,12,13,14,15,16,17,18]. One hundred and sixty-two epithets are listed under *Pezicula* in the Index Fungorum online database [1]. In recent years, morphological characters combined with PCR-based restriction fragment length polymorphism (RFLP) patterns of ribosomal DNA as well as DNA sequence analyses were used for reliable species identification. Forty-six species are currently recognized in the genus.

Phylogenetically, *Pezicula* is closely related to *Rhizodermea* Verkley & J.D. Zijlstra [5] and *Dermea* Fr. [19], and also associated with *Coleophoma* Höhn., *Davidhawksworthia* Crous, *Neofabraea* H.S. Jacks., *Parafabraea* Chen Chen, Verkley & Crous, *Phlyctema* Desm., and *Pseudofabraea* Chen Chen, Verkley & Crous based on sequence analyses of the internal transcribed spacer region (ITS), partial large subunit nrDNA (LSU), partial β-tubulin region (*tub2*), and partial RNA polymerase II second largest subunit (*rpb2*) regions [5,19,20,21,22]. The above-mentioned genera can be separated from each other by the morphology of sexual and/or asexual stages as well as DNA sequences.

In China, the first report of *Pezicula* species was attributed to Teng (1963) based on specimens of *P. ocellata* (Pers.) Seaver collected from Ningxia and Qinghai provinces [23]. Additional species have been recorded from different regions [7,9,12,13,24,25,26,27,28,29,30]. During our studies on the species diversity of Dermateaceae in China, herbarium specimens were re-examined, and recent collections of the genus were identified on the basis of morphological features and molecular data. The occurrence of 11 *Pezicula* species in China are summarized and reviewed based on the literature. In addition, two new species are described, and two new records are added to the fungal catalogue of China.

## 2. Materials and Methods

### 2.1. Morphological Observations

The study was based on newly collected materials and specimens preserved in the Herbarium Mycologicum Academiae Sinicae (HMAS). Macroscopic characters were recorded according to the field notes or observed under a SZX7 stereomicroscope (Olympus Corporation, Tokyo, Japan). Dried apothecia were rehydrated with distilled water and sectioned at a thickness of 15−20 μm with a YD-1508A freezing microtome (Yidi Medical Equipment Co., Ltd., Jinhua, Jiangsu, China). Iodine reactions of ascus apical apparatus were tested in Melzer’s reagent and Lugol’s solution with or without 3% KOH pretreatment [31]. Microscopic examinations and measurements were taken from dried apothecia under a BH-2 microscope (Olympus Corporation, Tokyo, Japan). Microscopic photographs were taken using an Axiocam 305 color microscope camera (Zeiss Corporation, Göttingen, Germany) attached to an Axioskop 2 Plus microscope (Zeiss Corporation, Göttingen, Germany).

### 2.2. DNA Extraction, PCR, and Sequencing

Genomic DNA was extracted from dried apothecia using a Plant Genomic DNA Kit (TIANGEN Biotech. Co., Beijing, China). PCR reaction mixtures had a final volume of 30 μL, containing 15 μL 2 × Taq MasterMix (CWBiotech. Co., Beijing, China), 1.5 μL of each primer (10 mM), 2 μL DNA, and 10 μL deionized water. The nuclear ribosomal internal transcribed spacer (ITS) region was amplified using the primers ITS1 and ITS4 [32]. PCR reactions were carried out in an Applied Biosystems 2720 thermocycler (Foster City, CA, USA) under the following conditions: 94 °C for 5 min, followed by 35 cycles of 94 °C for 30 s, 53 °C for 30 s and 30 s at 72 °C, and a final extension of 72 °C for 10 min. PCR products were purified and sequenced at Tianyi Huiyuan Bioscience and Technology Co. Ltd. (Beijing, China).

### 2.3. Sequence Assembly, Alignment, and Phylogenetic Analyses

Newly generated sequences were assembled using BioEdit 7.0.9.0 [33] and submitted to GenBank. ITS sequences of representative *Pezicula* species and two outgroup taxa were retrieved from GenBank (Table 1). The sequence dataset was aligned with MUSCLE [34] and manually adjusted where necessary in BioEdit 7.0.9.0 [33].

The ITS matrix was analyzed using Maximum Likelihood (ML) and Bayesian inference (BI) methods. ML analysis was conducted in RAxML 8.0 [35] using a GTRGAMMA model and tested with 1000 bootstrap replicates. BI analysis was performed using MrBayes 3.1.2 [36]. The best-fit nucleotide substitution model was estimated by MrModeltest 2.3 [37] with the Akaike information criterion. Two parallel runs of four simultaneous chains of Markov Chain Monte Carlo were performed for 2,000,000 generations with trees sampled every 100 generations. The first quarter of the sampled trees were discarded as the burn-in phase, and the remaining trees were used for calculating posterior probabilities (PP) in the majority rule consensus tree. The phylogenetic trees were visualized in FigTree v1.4.4 [38].

## 3. Results

### 3.1. Phylogeny

**Figure 1:**
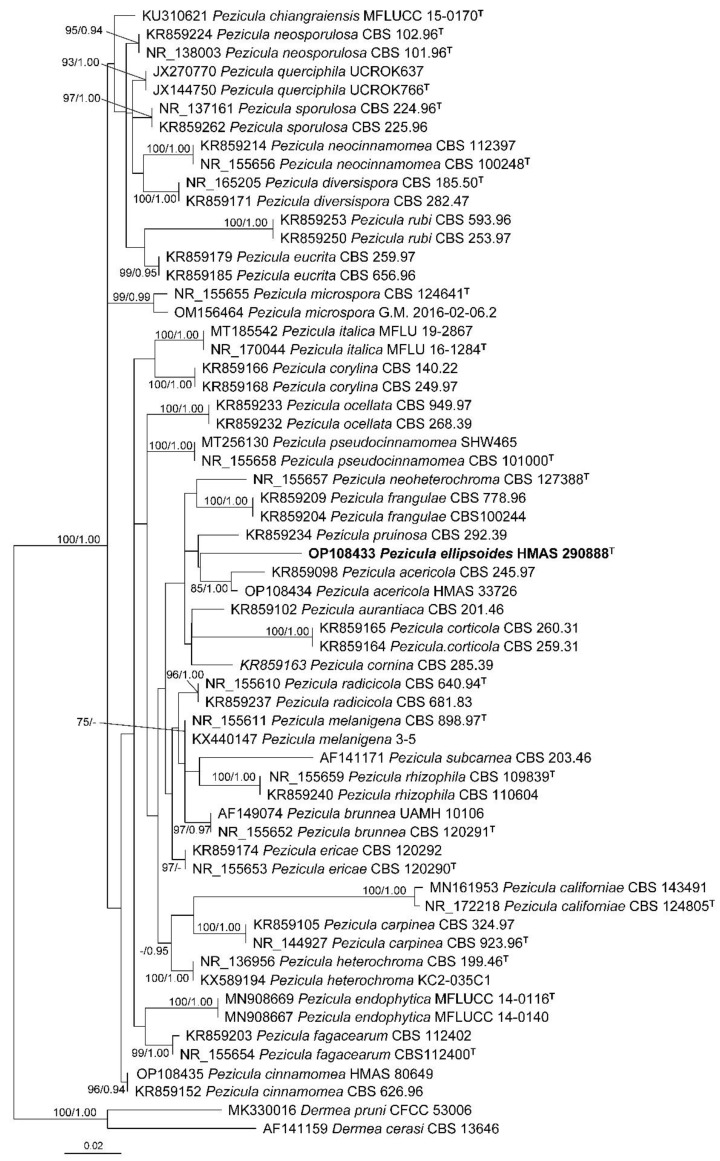
Maximum likelihood phylogenetic tree inferred from ITS sequences of *Pezicula* species. *Dermea cerasi* and *D. pruni* were used as outgroup taxa. Types are indicated with the superscript letter “T”. The new species proposed in the present study is in bold. Bootstrap support values ≥50% and Bayesian posterior probabilities ≥0.90 are shown at the nodes. The scale bar represents the number of nucleotide substitutions per site.

A total of 61 ITS sequences representing 33 *Pezicula* species and two outgroup taxa were included in the phylogenetic analyses (Table 1). The ITS alignment consisted of 542 characters including gaps (TreeBASE accession number: 29639). GTR+I+G was selected as the best-fit model. The resulting maximum likelihood phylogenetic tree is shown in Figure 1 with bootstrap support values of ML (≥50%) and Bayesian posterior probabilities (≥0.90) shown at the nodes from left to right. All the samples of *Pezicula* formed a well-supported monophyletic group, and all the investigated species were recognized.

### 3.2. Taxonomy

#### 3.2.1. New Species

**Figure 2:**
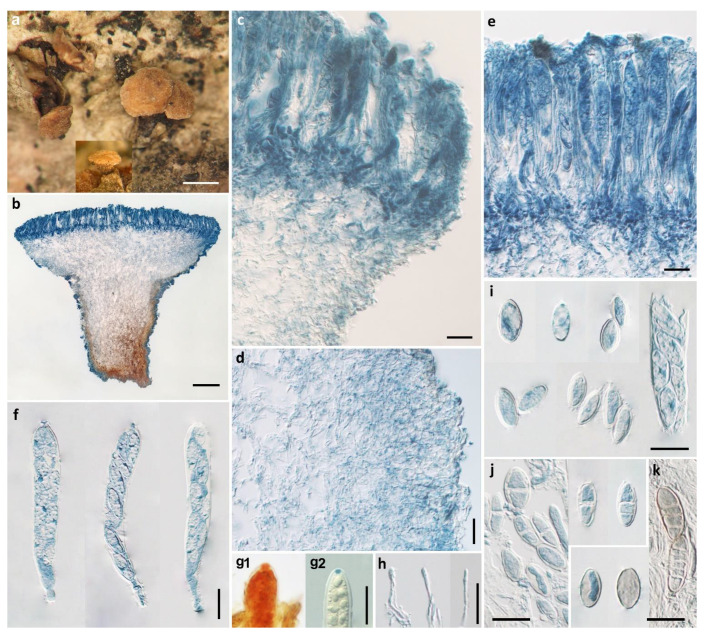
*Pezicula ellipsoides* H.D. Zheng & W.Y. Zhuang (HMAS 290888, holotype). (**a**) Dry apothecia on natural substrate; (**b**) longitudinal section of apothecium; (**c**) longitudinal section of apothecium (partial) showing excipular and hymenium structure at margin and upper flank; (**d**) excipular structure of stipe; (**e**) hymenium; (**f**) asci; (**g1**) IKI reaction of ascus apical ring without KOH pretreatment; (**g2**) IKI reaction of ascus apical ring after KOH pretreatment; (**h**) paraphyses; (**i**) ascospores; (**j**) ascospores with 0–1 septa; (**k**) light brown ascospores. Scale bars: (**a**) = 1 mm; (**b**) = 200 μm; (**c**–**k**) = 20 μm. Mounting media: (**b**–**f**, **h**–**k**) lactophenol cotton blue; (**g1**, **g2**) Lugol’s solution.

*Peziculaellipsoides* H.D. Zheng & W.Y. Zhuang, sp. nov. Figure 2.

Fungal Names: FN571258.

Etymology: The specific epithet refers to the ascospore shape of the species.

Holotype: CHINA, Hubei, Shennongjia, alt. 2500 m, on rotten bark, 14 September 2014, H.D. Zheng et al. 9542, HMAS 290888.

Apothecia erumpent, solitary or 2–3 on a basal stroma, discoid with surface convex, margin entire, short stipitate, 1–2 mm in diam; hymenium light yellow when fresh, pale brownish-yellow when dry; receptacle surface concolorous. Ectal excipulum of textura angularis, 50–80 μm thick, cells hyaline to light brown, isodiametric or nearly so, 4–16 μm in diam. Medullary excipulum of textura intricata, 50–300 μm thick, hyphae hyaline, 3.5–6 μm wide, becoming light brown in stipe and some swollen up to 20 μm in diam. Subhymenium hyaline, composed of interwoven hyphae, about 40 μm thick. Hymenium 140–150 μm thick. Asci arising from simple septa, 8-spored, cylindrical-clavate, with apex rounded to conical-rounded, narrowed gradually or abruptly into a short stalk, apical rings J+, becoming blue only after KOH pretreatment in Melzer’s reagent and not changing color without KOH pretreatment, and becoming red without KOH pretreatment and blue after KOH pretreatment in Lugol’s solution, 110–128 × 15–18 μm. Ascospores ellipsoid, with ends rounded or somewhat pointed, 0–1(–2)-septate, tardily becoming 3–5-septate, thin-walled, hyaline to light brown, multiguttulate, irregularly uniseriate in asci, 15.5–22.5 × 7.5–12 μm. Paraphyses filiform, septate, simple, hyaline, 2–2.5 μm wide, slightly enlarged at apex, equaling to or slightly exceeding the asci.

Notes: In the BLASTN search of the ITS region of *P. ellipsoides* in NCBI, *P. cinnamomea* (CBS 242.60, MH857970) shares the highest sequence similarity of 96.55% (503/521), followed by *P. brunnea* (CBS 120291, NR_155652) and *P. acericola* (CBS 248.97, KR859101), both with 96.35% (502/521) similarity. The phylogenetic analyses confirmed the placement of *P. ellipsoides* in *Pezicula* and its distinction from any other species of the genus.

*Pezicula puberula* (E.J. Durand) Verkley is similar to *P. ellipsoides* in the morphology of apothecia, asci, and ascospores, but differs in the presence of its whitish pruinate hymenium surface, up to 15 apothecia attached to a single basal stroma, cells of ectal excipulum 10–25 × 5–8 μm, and receptacle surface covered by amorphous crystals [2]. The ascospores of *P. ellipsoides* resemble those of *P. sepium* (Desm.) Dennis; however, the latter possesses smaller (less than 1.4 mm in diam), sessile to subsessile apothecia, thick-walled medullary excipular cells, 7–12 × 5–8 μm, and larger asci, 130–165 × 15–23 μm [2].

**Figure 3:**
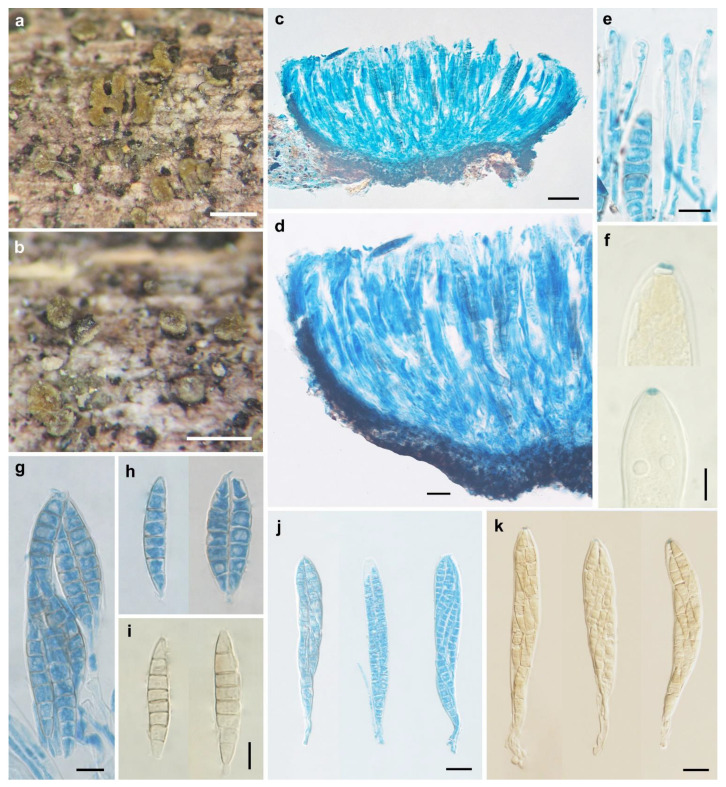
*Pezicula fusispora* W.Y. Zhuang & H.D. Zheng (HMAS 290892, holotype). (**a**,**b**) Dry apothecia on natural substrate; (**c**) longitudinal section of apothecium; (**d**) longitudinal section of apothecium (partial) showing excipular and hymenium structure; (**e**) paraphyses; (**f**) IKI reaction of ascus apical ring without KOH pretreatment; (**g**–**i**) ascospores; (**j**,**k**) asci. Scale bars: (**a**,**b**) = 0.5 mm; (**c**) = 50 μm; (**d**,**j**,**k**) = 20 μm; (**e**–**i**) = 10 μm. Mounting media: (**c**–**e**,**g**,**h**,**j**) lactophenol cotton blue; (**f**,**i**,**k**) Lugol’s solution.

*Pezicula fusispora* W.Y. Zhuang & H.D. Zheng, sp. nov. Figure 3.

Fungal Names: FN571257.

Etymology: The specific epithet refers to the fusoid ascospores of the fungus.

Holotype: CHINA, Hunan, Yizhang, Mangshan, alt. 1200 m, on rotten bamboo, 11 April 2002, W.Y. Zhuang, B. Liu & X.Z. Liu 4162, HMAS 290892.

Apothecia slightly erumpent, solitary or 2–5 on a basal stroma, discoid, sessile; disc circular, elliptical to irregular, slightly convex, concave when dry, hymenium is bright yellow to yellowish-brown, dull yellow with olivaceous tint when dry, 0.3–0.8 mm in diam; receptacle near and at the margin concolorous with the hymenium, brownish at flanks and towards the base; margin entire or slightly torn. Ectal excipulum of textura angularis, 20–30 μm thick, cells brown, isodiametric or nearly so, 5–12 × 4–10 μm. Medullary excipulum of textura intricata, poorly developed, 30–40 μm thick, hyphae hyaline, 2–3 μm in diam. Subhymenium indistinguishable. Hymenium 155–175 μm thick. Asci arising from simple septa, 8-spored, cylindrical-clavate, with apex conical-rounded, narrowed gradually into a short, thin stalk, apical rings J+, blue in Melzer’s reagent and Lugol’s solution without KOH pre-treatment, 135–170 × 15–21 μm. Ascospores fusoid, with ends pointed, inequilateral, 3–7-septate, thin-walled, hyaline to light brown, multiguttulate, irregularly biseriate in asci, 33–48 × 7–10.3 μm. Paraphyses filiform, septate, simple, hyaline, 2.5–3 μm wide, slightly enlarged at apex, equaling to or slightly exceeding the asci.

Other specimen examined: CHINA, Hunan, Nanyue, Zhurongfeng, alt. 700 m, on rotten bamboo, 11 April 2002, W.Y. Zhuang 4136, HMAS 290893.

Notes: *Pezicula fusispora* can be easily distinguished by the combination of sessile apothecia with bright yellow to yellowish-brown hymenium, J+ asci in iodine solutions, and multiseptate ascospores which are fusoid and 33–48 × 7–10.3 μm in size. *Pezicula aurantiaca* and *P. ocellata* also have sessile apothecia but are different in hymenium color, J− ascus apical apparatus, and ascospores that are ellipsoid, smaller, and with fewer septa [2]. Unfortunately, no DNA sequence was retrieved from the voucher specimens, and the phylogenetic position of the fungus within the genus awaits future investigation.

#### 3.2.2. New Records for China

**Figure 4:**
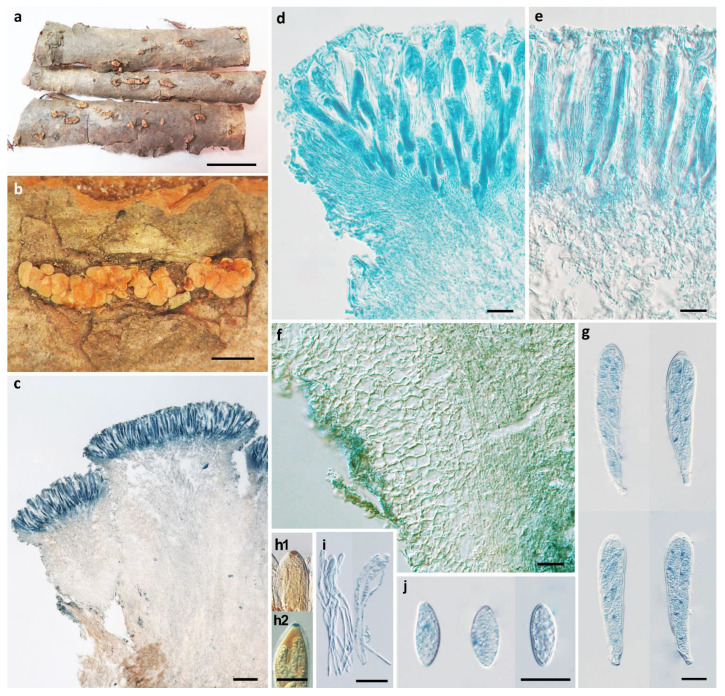
*Pezicula acericola* (Peck) Peck ex Sacc. & Berl. (HMAS 33726). (**a**,**b**) Dry apothecia on natural substrate; (**c**) longitudinal section of apothecia; (**d**) longitudinal section of apothecium (half) showing hymenium and excipular structure; (**e**) hymenium; (**f**) excipular structure at flank; (**g**) asci; (**h1**) IKI reaction of ascus apical ring without KOH pretreatment; (**h2**) IKI reaction of ascus apical ring after KOH pretreatment; (**i**) paraphyses; (**j**) ascospores. Scale bars: (**a**) = 2 cm; (**b**) = 2 mm; (**c**) = 100 μm; (**d**–**j**) = 20 μm. Mounting media: (**c**–**g**,**i**,**j**) lactophenol cotton blue; (**h1**,**h2**) Lugol’s solution.

*Pezicula acericola* (Peck) Peck ex Sacc. & Berl., Atti Inst. Veneto Sci. Lett., ed Arti (Sér. 6) 3: 725, 1885. Figure 4.

≡*Nodularia acericola* Peck, Ann. Rep. N.Y. St. Mus. 24: 98, 1872.

Apothecia erumpent, a few to a large number in circular to linear clusters on a basal stroma, discoid with surface plane to slightly convex, margin entire or irregularly torn, short stipitate; hymenium yellow when fresh, light cinnamon when dry, 0.3–3 mm in diam; receptacle concolorous to paler than hymenium. Ectal excipulum of textura angularis, 25–180 μm thick, cells subhyaline to light brown, isodiametric or nearly so, 4–16.5 μm in diam. Medullary excipulum of textura intricata, 20–190 μm thick, hyphae hyaline to light brown, 4–8.5 μm wide. Subhymenium indistinguishable. Hymenium 125–150 μm thick. Asci arising from croziers, clavate, apex conical-rounded, narrowed gradually or abruptly into a short stalk, 8-spored, apical rings J+, becoming blue only after KOH pretreatment in Melzer’s reagent, and becoming red without KOH pretreatment and blue after KOH pretreatment in Lugol’s solution, 98.5–126 × 16.5–22 μm. Ascospores ellipsoid with ends narrower, slightly inequilateral, 0(–3)-septate, thin-walled, hyaline, multiguttulate, irregularly biseriate in asci, 24.5–33 × 7.5–11 μm. Paraphyses filiform, septate, hyaline, slightly swollen to 3–5 μm wide at apex and 1.5–3 μm wide below, equaling to or slightly exceeding the asci.

Specimens examined: China, Jilin, Antu County, Mt. Changbai, alt. 1600 m, on rotten twig, 11 August 1960, Y.C. Yang, J.R. Li & F.S. Yuan 673 (HMAS 33726); Jilin, Antu County, Mt. Changbai Forestry Station, alt. 1750 m, on rotten bark, 25 July 1960, Y.C. Yang, J.R. Li & F.S. Yuan 420 (HMAS 33749).

Known distribution: Asia (China, Japan), Europe (Austria, Czech Republic, France, Italy, the Netherlands, Sweden, Switzerland, United Kingdom), and North America (Canada, USA^#^) (^#^ indicating country of type locality).

Notes: *Pezicula acericola* is widely distributed in Europe and North America [2]. The macroscopic and microscopic features of the Chinese materials coincide with those from other countries. The ITS sequence of the Chinese collection (HMAS 33726) has a 98.89% similarity with those of the Canadian materials (KR859094-KR859101). This is a new record for China. The two voucher specimens (HMAS 33726 and HMAS 33749) were originally misidentified as *P. aurantiaca*, which is clearly different in possessing sessile apothecia that are mostly solitary or 2–3(–4) on a basal stroma, asci J− in Melzer’s reagent, and ascospores smaller in size (14.5–23 × 6–10 μm) [2].

**Figure 5:**
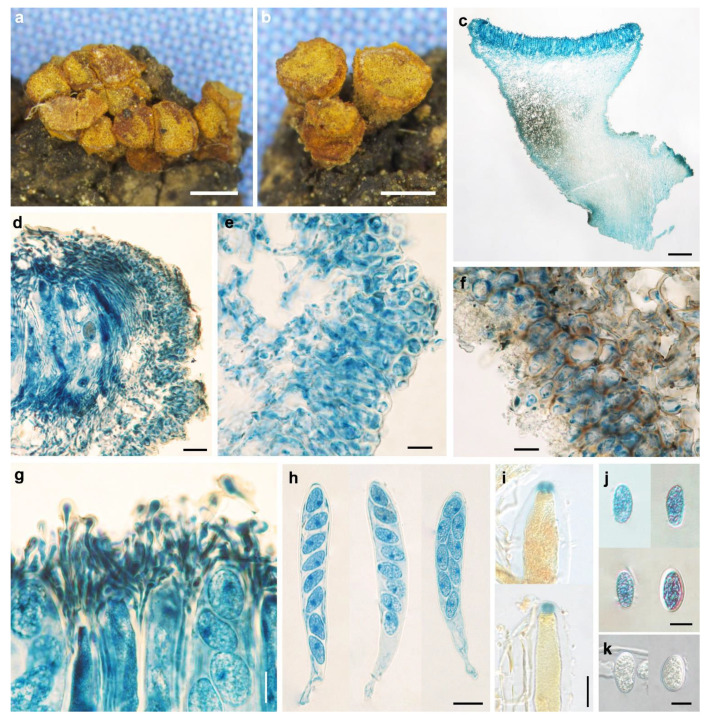
*Pezicula carpinea* (Pers.) Tul. ex Fuckel (HKAS 5295). (**a**,**b**) Dry apothecia on natural substrate; (**c**) longitudinal section of apothecium; (**d**) excipular structure of margin; (**e**) excipular structure of flank; (**f**) excipular structure adjacent to flank and stipe; (**g**) apices of asci and paraphyses; (**h**) asci; (**i**) IKI reaction of apical ring; (**j**,**k**) ascospores. Scale bars: (**a**,**b**) = 1 mm; (**c**) = 200 μm; (**d**,**h**) = 20 μm; (**e**–**g**,**i**–**k**) = 10 μm. Mounting media: (**c**–**h**,**j**) lactophenol cotton blue; (**i**) Lugol’s solution; (**k**) 3% KOH.

*Pezicula carpinea* (Pers.) Tul. ex Fuckel, Jb. Nassau. Ver. Naturk. 23–24: 279, 1870. Figure 5.

≡*Peziza carpinea* Pers., Syn. Meth. Fung. (Göttingen) 2: 673, 1801.

Apothecia erumpent, mostly 2–15 on a basal stroma, discoid, margin slightly raised, drying concave, substipitate to short-stipitate, 0.5–2.0 mm in diam; hymenium pruinose, ochraceous when dry; receptacle concolorous with hymenium. Ectal excipulum of textura angularis to textura globulosa, 25–40 μm thick, cells hyaline to brown, isodiametric or nearly so, axis of cells arranged at a high to right angle to the outer surface, 5–20 μm in diam. Medullary excipulum of textura intricata, 110–220 μm thick, hyphae hyaline to light brown, 4–5.5 μm wide. Subhymenium composed of interwoven hyphae, 40–55 μm thick, cells hyaline. Hymenium 150–165 μm thick. Asci arising from simple septa, cylindrical-clavate, apex rounded to truncate-rounded, narrowed gradually into a stalk of varying length, 8-spored, apical rings J+, becoming blue in Melzer’s reagent and Lugol’s solution without KOH pre-treatment, 129–165 × 21–25 μm. Ascospores ellipsoid to broadly ellipsoid, slightly inequilateral, with ends rounded, nonseptate, thin-walled, hyaline, multiguttulate, uniseriate to biseriate in asci, 19.2–24.6 × 10.5–11.5 μm. Paraphyses filiform, septate, simple or branched at upper part, hyaline to very pale brown, swollen to 3.5–5 μm at apex and 2–3 μm wide below, exceeding the asci by 15–25 μm.

Specimen examined: China, Tibet, Longzhan, Gibala, on rotten wood, 16 July, 1975, M. Zang 295 (HKAS 5295).

Known distribution: Asia (China), Europe (Austria, Czech Republic, France, Germany^#^, Hungary, the Netherlands, Slovenia, Sweden, United Kingdom), North America (Canada, USA).

Notes: *Pezicula carpinea* is the type species of the genus, which was previously reported from Europe and North America [2,5]. The specific epithet of the species refers to its common occurrence on *Carpinus* sp. The sexual and asexual characteristics of the species have been well-documented and illustrated in previous studies [2,5]. The features of the Chinese collection are concordant with those from other regions of the world. The overmatured ascospores were recorded as yellow-brown and muriform [2], but such ascospores were not found in the Chinese material due to the different stage of development.

#### 3.2.3. Species Previously Recorded from China

*Pezicula cinnamomea* (DC.) Sacc., Syll. Fung. 8: 311, 1889.

≡*Peziza cinnamomea* DC., Fl. franç., Edn 3 (Paris) 5/6: 23, 1815.

Specimen examined: China, Guangxi, Wuming, Mt. Daming, alt. 1200 m, on rotten twig of a conifer tree, 22 December 1997, W.P. Wu & W.Y. Zhuang 1902 (HMAS 74848).

Known distribution: Asia (China, Japan,), Europe (Austria, Belgium, Czech Republic, Denmark, France^#^, Germany, Netherlands, Norway, Russia, Spain, Sweden, Switzerland, United Kingdom), North America (Canada, USA), Central America (Costa Rica), Oceania (New Zealand).

Notes: The species was reported from Guangxi Province in China [26]. It occurs on broad-leaved trees as well as conifers [2].

*Pezicula ericae* (Sigler) P.R. Johnst., in Johnston, Seifert, Stone, Rossman & Marvanová, IMA Fungus 5(1): 104, 2014.

≡*Cryptosporiopsis ericae* Sigler, in Sigler, Allan, Lim, Berch & Berbee, Stud. Mycol. 53: 57, 2005.

Known distribution: Asia (China), Europe (Norway), North America (USA^#^).

Notes: *Pezicula ericae* was originally described in the USA as an endophytic fungus from roots of ericaceous plants [10]. Subsequently, strains of the species were isolated from orchids [39,40], aspen [11], pine [41], spruce [42], fir [43], and other hosts [6,44], in addition to ericaceous plants [45,46,47]. The species is able to promote plant growth [48], improve the drought resistance ability of plants [49], and synthesize compounds with antimicrobial activities against phytopathogenic bacteria and fungi [6,44].

*Pezicula heterochroma* Verkley, Stud. Mycol. 44: 94, 1999.

Known distribution: Asia (China), North America (Canada^#^).

Notes: The type of the species grew on recently dead bark of *Alnus mollis* in Canada [2]. The Chinese materials were recorded as an endophyte of *Vaccinium dunalianum*, with strong antagonistic effects against a wood-decaying fungus, *Poria placenta* (Fr.) Cooke (≡ *Rhodonia placenta* (Fr.) Niemelä, K.H. Larss. & Schigel) [30].

*Pezicula magnispora* Z.H. Zhong & Zheng Wang, in Zhong, Wang & Pfister, Mycotaxon 78: 161, 2001.

Specimen examined: China, Sichuan, Luding, Mt. Gongga, alt. 1200 m, on herbaceous stem, 19 August 1997, Wang Z. 2097 (holotype, HMAS 75908).

Known distribution: Asia (China^#^).

Notes: The species was originally described from Sichuan Province and is distinctive in producing large, muriform ascospores (46–58 × 14.5–20 μm) and growing on herbaceous stems [12]. Further distribution was not reported. The type specimen of *P. magnispora* is too scanty to extract DNA, and sequence data are not available.

*Pezicula melanigena* (T. Kowalski & Halmschl.) P.R. Johnst., in Johnston, Seifert, Stone, Rossman & Marvanová, IMA Fungus 5(1): 104, 2014.

≡*Cryptosporiopsis melanigena* T. Kowalski & Halmschl. (as ‘*Cryptosporioposis*’), in Kowalski, Halmschlager & Schrader, Mycol. Res. 102(3): 348, 1998.

Known distribution: Asia (China), Europe (Austria^#^).

Notes: *Pezicula melanigena* was originally isolated from roots of healthy and declining 80–120-yr-old oak trees (*Quercus robur* and *Q. petraea*) in Austria and is only known from its asexual stage. It was also stated that the intensity of culture pigmentation, growth rate, size of macroconidia, color of chlamydospores, and shape of setae are variable among isolates of the species [50]. The fungus was detected in forest soil samples from northeastern China via a high-throughput sequencing method [28].

*Pezicula neosporulosa* Z.L. Yuan & Verkley, Mycoscience 56: 206, 2014.

Known distribution: Asia (China, Korea), Europe (the Netherlands^#^), Oceania (New Zealand).

Notes: The species was originally introduced to science based on the materials from the Netherlands and China [13], and the Chinese samples were all endophytes of *Abies beshanzuensis* from Zhejiang Province. Subsequently, a strain was isolated from soil in Korea [51]. It is also known as an endophytic fungus of *Pseudowintera colorata*, an endemic medicinal plant of New Zealand [52]. The strain VDBF-2 isolated from *Vaccinium dunalianum* in Yunnan Province showed strong antagonistic effects against plant pathogens and wood-decaying fungi [30]. Based on comparative and pan-genomic analyses of the endophytic *P. neosporulosa*, an example of convergent genomic adaptation to endophytism was revealed, mostly determined by enzymes involved in carbohydrate metabolism and secondary metabolite biosynthesis [53].

*Pezicula ocellata* (Pers.) Seaver, North American Cup-fungi, (Inoperculates) (New York): 345, 1951.

≡*Peziza ocellata* Pers., Syn. Meth. Fung. (Göttingen) 2: 667, 1801.

Specimens examined: China, Ningxia, Mt. Helan, alt. 2600 m, on rotten twig, 23 June 1961, S.J. Han, Q.M. Ma & Y. Liu 2200 (HMAS 32060); Qinghai, Qilian, Ladonggou, alt. 2800 m, on living bark of ?*Salix pseudotangii* C. Wang & C.Y. Yu, 18 Aug. 1958, Q.M. Ma 521 (HMAS 33643).

Known distribution: Asia (China). Europe (Czech Republic, Denmark, France^#^, Germany, Luxembourg, Norway, Poland, Sweden, UK), North America (Canada).

Notes: *Pezicula ocellata* is prominent in its sessile and orange to orange-brown apothecia with a white margin [2], and the color illustrations of the sexual and asexual stages were provided by Chen et al. (2016) [5].

*Pezicula rhizophila* (Verkley & J.D. Zijlstra) P.R. Johnst., in Johnston, Seifert, Stone, Rossman & Marvanová, IMA Fungus 5(1): 104, 2014.

≡*Cryptosporiopsis rhizophila* Verkley & J.D. Zijlstra, in Verkley, Zijlstra, Summerbell & Berendse, Mycol. Res. 107(6): 694, 2003.

Known distribution: Asia (China), Europe (the Netherlands^#^).

Notes: *Pezicula rhizophila* is known as an endophytic fungus of several ericaceous plants [54]. An endophytic strain FLR13 of the species was isolated from roots of cultivated blueberry in China, which showed a strong antagonistic ability against five plant pathogens, the ability of plant growth enhancement, and its application potential as a biocontrol agent in agriculture [7]. The genome data of the strain were available in the NCBI database (Accession: JAJONI000000000), for which no relevant paper has been published.

*Pezicula* cf. *rubi* (Lib.) Niessl, in Rabenhorst, Fungi Europ. Exsicc. no. 2122. 1876.

≡*Patellaria rubi* Lib., Pl. crypt. Arduenna, fasc. (Liège) 3(nos 201–300): no. 231, 1834.

Specimen examined: China, Yunnan, Xishuangbanna, on bark of *Tetrastigma* sp., 22 October 1988, Korf, Zang, Chen & W.Y. Zhuang 229 (HMAS 72156).

Known distribution: Asia (China), Europe (Austria, Belgium^#^, Czech Republic, Denmark, France, Germany, Italy, Netherlands, Sweden, UK), North America (Canada).

Notes: *Pezicula rubi* normally grows on *Rubus* or *Rosa*, while the natural substrate of the Chinese collection was recorded as *Tetrastigma* sp. [25] which is not a common substrate for the fungus.

*Pezicula subcarnea* J.W. Groves, Mycologia 33: 517. 1941.

Known distribution: Asia (China), North America (Canada^#^, USA).

Notes: The species was originally reported from Canada growing on *Acer pennsylvanicum*, and the sexual and asexual stages were described and illustrated in detail [55]. In China, it was only reported from Taiwan Province [24].

#### 3.2.4. Species Excluded from the Fungal Catalogue of China

*Pezicula aurantiaca* (Rehm) Rehm, Ber. Bayer. Bot. Ges. 13: 198, 1912.

≡*Habrostictis aurantiaca* Rehm, Ber. naturhist. Augsburg 26: 67, 1881.

Notes: *Pezicula aurantiaca* was originally described from Austria and also known from Canada [2]. The species was reported from China based on two specimens [21], which were reidentified as *P. acericola* based on morphological features as well as DNA sequence data. Thus, *P. aurantiaca* should be excluded from the fungal catalogue of China [29].

## 4. Discussion

In this study, two novel species and two new Chinese records of *Pezicula* were discovered based on morphological features, and the positions of two of them, *P. ellipsoides* and *P. acericola*, were further confirmed by phylogenetic inference. Eleven species of the genus previously recorded from China were reviewed, of which one misidentified name should be excluded. Up to now, 14 *Pezicula* species are known from the country, among which nine were known as saprobes [12,23,24,25,26], four were plant endophytes [7,13,30,45], and one was even reported in soil [28] but might also be plant-associated.

As far as the lifestyles of *Pezicula* species are concerned, they are supposed to be initially endophytic in living plant tissues but switch to saprophytic when their hosts die, and those which are weakly phytopathogenic may cause diseases when their hosts are under stress [2,5]. Some of the endophytic species show antifungal effects against plant pathogens, some may produce antibiotics and fungicidal or herbicidal compounds [2,4,6,7,30,56,57,58,59], and others possess the ability to promote plant growth [7,48]. In view of the antimicrobial activity and plant growth promotion of *Pezicula*, they might have application potential in plant disease control and increasing crop yield.

In most cases, morphological characteristics are sufficient to distinguish *Pezicula* species, especially those that have both sexual and asexual stages. DNA sequence data are very helpful in discovering new species and understanding relationships among taxa within the group. ITS, LSU, *rpb2*, *tub2*, and translation elongation factor-1 alpha (*tef-1α*) gene sequences have been used in the taxonomic and phylogenetic studies of the genus [5,13,14,18]. Based on the results of the previous studies and our analyses, the variations of the ITS region appear to be able to distinguish *Pezicula* species correctly. Additional research should be carried out regarding species diversity, the connection of sexual and asexual stages, the selection of supplementary DNA barcodes, and the exploration of potential uses of the members of the genus. Knowing the species diversity is the first step in gaining this knowledge. We expect to find additional *Pezicula* species in our future surveys in different regions of the country.

## 5. Conclusions

The species diversity of the genus *Pezicula* in China was explored. Two new species and two new Chinese records of the genus were discovered, described, and illustrated. The previously reported taxa were reviewed with one excluded from the fungal catalogue of China, resulting in 14 species that are currently known from the country.

## Figures and Tables

**Table 1:** List of taxa and GenBank accession numbers of ITS sequences used in the study.

Species	Strain/Voucher Specimen	Locality	ITS
*Dermea cerasi* (Pers.) Fr.	CBS 136.46	USA	AF141159
*D. pruni* (Teng) J.W. Groves	CFCC 53006	China	MK330016
*Pezicula acericola* (Peck) Peck ex Sacc. & Berl.	CBS 245.97	Canada	KR859098
*P. acericola*	HMAS 33726	China	OP108434 **
*P. aurantiaca* (Rehm) Rehm	CBS 201.46	Canada	KR859102
*P. brunnea* (Sigler) P.R. Johnst.	CBS 120291**^T^**	Canada	NR_155652
*P. brunnea*	UAMH 10106		AF149074
*P. californiae* (Cheew., Denman & Crous) P.R. Johnst.	CBS 124805**^T^**	USA	NR_172218
*P. californiae*	CBS 143491		MN161953
*P. carpinea* (Pers.) Tul. ex Fuckel	CBS 923.96**^T^**	Germany	NR_144927
*P. carpinea*	CBS 324.97	The Netherlands	KR859105
*P. chiangraiensis* Ekanayaka & K.D. Hyde	MFLUCC 15-0170**^T^**	Thailand	KU310621
*P. cinnamomea* (DC.) Sacc.	CBS 239.96	France	KR859124
*P. cinnamomea*	HMAS 80649	The Netherlands	OP108435 **
*P. cornina* (Peck) P.R. Johnst.	CBS 285.39	Canada	KR859163
*P. corticola* (C.A. Jørg.) Nannf.	CBS 259.31	Denmark	KR859164
*P. corticola*	CBS 260.31	Denmark	KR859165
*P. corylina* J.W. Groves	CBS 140.22	UK	KR859166
*P. corylina*	CBS 249.97	Canada	KR859168
*P. diversispora* (Robak) P.R. Johnst.	CBS 185.50**^T^**	Norway	NR_165205
*P. diversispora*	CBS 282.47		KR859171
*P. ellipsoides* H.D. Zheng & W.Y. Zhuang	HMAS 290888**^T^**	China	OP108433 **
*P. endophytica* X.Y. Ma, K.D. Hyde & J.C. Kang	MFLUCC 14-0116**^T^**		MN908669
*P. endophytica*	MFLUCC 14-0140		MN908667
*P. ericae* (Sigler) P.R. Johnst.	CBS 120290**^T^**	USA	NR_155653
*P. ericae*	CBS 120292	Canada	KR859174
*P. eucrita* (P. Karst.) P. Karst.	CBS 259.97	USA	KR859179
*P. eucrita*	CBS 656.96	Germany	KR859185
*P. fagacearum* Chen Chen, Verkley & Crous	CBS 112400**^T^**	Italy	NR_155654
*P. fagacearum*	CBS 112402	Italy	KR859203
*P. frangulae* (Fr.) Fuckel	CBS 778.96	The Netherlands	KR859209
*P. frangulae*	CBS 100244	Denmark	KR859204
*P. heterochroma* Verkley	CBS 199.46**^T^**	Canada	NR_136956
*P. heterochroma*	KC2-035C1		KX589194
*P. italica* W.J. Li, Camporesi & K.D. Hyde	MFLU 16-1284**^T^**	Italy	NR_170044
*P. italica*	MFLU 19-2867	Italy	MT185542
*P. melanigena* (T. Kowalski & Halmschl.) P.R. Johnst.	CBS 898.97**^T^**	Austria	NR_155611
*P. melanigena*	3–5		KX440147
*P. microspora* Chen Chen, Verkley & Crous	CBS 124641**^T^**	Italy	NR_155655
*P. microspora*	G.M. 2016-02-06.2		OM156464
*P. neocinnamomea* Chen Chen, Verkley & Crous	CBS 100248**^T^**	Denmark	NR_155656
*P. neocinnamomea*	CBS 112397		KR859214
*P. neoheterochroma* Chen Chen, Verkley & Crous	CBS 127388**^T^**	Austria	NR_155657
*P. neosporulosa* Z.L. Yuan & Verkley	CBS 101.96**^T^**	The Netherlands	NR_138003
*P. neosporulosa*	CBS 102.96**^T^**	The Netherlands	KR859224
*P. ocellata* (Pers.) Seaver	CBS 949.97	Luxembourg	KR859233
*P. ocellata*	CBS 268.39	Germany	KR859232
*P. pruinosa* Farl.	CBS 292.39	Canada	KR859234
*P. pseudocinnamomea* Chen Chen, Verkley & Crous	CBS 101000**^T^**	The Netherlands	NR_155658
*P. pseudocinnamomea*	SHW465		MT256130
*P. querciphila* (S.C. Lynch, Eskalen & Zambino) P.R. Johnst.	UCROK766**^T^**	USA	JX144750
*P. querciphila*	UCROK637	USA	JX270770
*P. radicicola* (Kowalski & Bartnik) P.R. Johnst.	CBS 640.94**^T^**	Poland	NR_155610
*P. radicicola*	CBS 681.83	Austria	KR859237
*P. rhizophila* (Verkley & J.D. Zijlstra) P.R. Johnst.	CBS 109839**^T^**	The Netherlands	NR_155659
*P. rhizophila*	CBS 110604	The Netherlands	KR859240
*P. rubi* (Lib.) Niessl	CBS 253.97	USA	KR859250
*P. rubi*	CBS 593.96	The Netherlands	KR859253
*P. sporulosa* Verkley	CBS 224.96**^T^**	The Netherlands	NR_137161
*P. sporulosa*	CBS 225.96**^T^**	The Netherlands	KR859262
*P. subcarnea* J.W. Groves	CBS 203.46	Canada	AF141171

Newly generated sequence data are marked with **. Types are indicated with the superscript “T”.

## Data Availability

Names of the new species were formally registered in the database Fungal Names (https://nmdc.cn/fungalnames, accessed on 1 August 2022). Specimens were deposited in the Herbarium Mycologicum Academiae Sinicae (HMAS). The newly generated sequences were deposited in GenBank (https://www.ncbi.nlm.nih.gov/genbank, accessed on 1 August 2022). Alignment and trees were submitted to TreeBase (https://www.treebase.org, accessed on 6 September 2022).
